# Magnetic Properties and Corrosion Resistance of Sintered Nd-Fe-B Magnet Caused by Er_69_Fe_31_ Alloy Grain Boundary Addition

**DOI:** 10.3390/ma18122711

**Published:** 2025-06-09

**Authors:** Yongtao Dai, Kai Wang, Jing Xiang, Qingrong Yao, Zhao Lu, Jiang Wang

**Affiliations:** 1 Fujian Golden Dragon Rare-Earth Co., Ltd., Longyan 366399, China; daiytmtr@gmail.com; 2 Guangxi Key Laboratory of Information Materials, Guangxi Collaborative Innovation Center of Structure and Property for New Energy Materials, Guilin University of Electronic Technology, Guilin 541004, China; luzhao_gx@163.com (Z.L.); waj124@guet.edu.cn (J.W.); 3 Guangxi Key Laboratory of Marine Environmental Science, Guangxi Academy of Sciences, Nanning 530007, China

**Keywords:** Er_69_Fe_31_ alloy, sintered Nd–Fe–B magnet, corrosion resistance, magnetic properties

## Abstract

This work reports on the effect of the heavy rare earth element Er on Nd–Fe–B magnets by using a simple Er_69_Fe_31_ alloy additive, which is much less expensive than Dy and Tb elements. It was found that the corrosion resistance was improved with a minimal reduction in magnetic properties by rationally controlling the Er_69_Fe_31_ addition content. The main reason is that Er element partially replaces the Nd element at the edge of the main phase grain to form an (Er,Nd)_2_Fe_14_B shell with low *H*_A_, which leads to a decrease in coercivity. However, the improvement in the corrosion resistance is mainly due to the Er_69_Fe_31_ alloy addition, which slows down the corrosion rate. Simultaneously, an investigation was conducted into the different advantages that target magnets when subjected to diverse heat treatment methodologies. The above findings may lead to the development of applications for other rare earth elements, thereby accelerating the development of low-cost permanent magnets comparable to commercially available sintered Nd-Fe-B magnets.

## 1. Introduction

With the gradual implementation of scientific and technological progress and development, the market share of new energy materials is increasing. Among them, the demand for rare earth permanent magnet materials [1,2], which occupy an important position in the field of new energy, is also gradually increasing. The demand for cost-effective magnetic materials has been increasing, leading to the rapid occupation of the rare earth permanent magnetic materials market by sintered Nd–Fe–B magnets with higher magnetic properties and a lower cost [3,4,5,6]. However, the high intensity of the exploitation of rare earth resources, the huge market demand, and the low utilization of rare earth elements in corporate production limit the development of rare earth elements [7,8]. To cope with this unfavourable situation, various changes to the manufacturing process are necessary.

Grain boundary addition technology mainly involves melting a selected low-melting-point auxiliary alloy into a liquid phase during the sintering process [9]. This not only improves the wettability of the main phase and grain boundary phases but also leads to a more homogeneous distribution of the grain boundary phases, resulting in effective segregation [10,11]. The main phase promotes the transformation of the grain boundary phase from the bulk grain boundary phase into the network and strip grain boundary phase, densifying the magnet. In recent years, a significant number of studies have been conducted. Sun et al. [12] found that Ho–Fe alloys can improve the magnetic properties and greatly improve the utilization of rare earth elements by using grain boundary reconstruction. Because of their low melting point, (Ho–Fe alloys) will melt into the liquid phase during the sintering process, so the advantages of liquid phase sintering can minimise (reduce) defects, such as porosity, inside the magnets. Zhong et al. [13] reported that the coercivity temperature stability of magnets is favoured when the Y element partially replaces Pr and Nd elements. This occurs because Y element substitution exerts a distinct grain-refining effect on the main phase, and this resultant grain size refinement enhances intergranular exchange coupling, which directly accounts for the observed increases in coercivity and thermal stability [14,15]. Similarly, we wondered whether the rare earth element Er could also have a positive effect like Ho or Y elements. Although Er atoms can form 2:14:1 compounds like Nd_2_Fe_14_B, their multi-faceted (other) nature makes them less useful in sintered Nd-Fe-B magnets. If the amount of Er element addition can be controlled in an appropriate range and can play a positive role, then there would also be a balanced use of rare earth element resources. Based on the above idea, we investigated whether the Er element also has an effect similar to the heavy rare earth elements Dy and Tb so as to achieve the microsubstitution of the valuable heavy rare earth elements Dy and Tb [16,17].

Therefore, building on our previous work on the low-melting-point eutectic alloy Er_30_Cu_70_ [18], the low-melting-point eutectic alloy Er_69_Fe_31_ was introduced to exclude the influence of Cu elements through grain boundary reconstruction in order to study the effects of the Er element on the microstructure and properties of a target magnet in this study. Furthermore, the results will provide parametric guidance for optimising the distribution and rational use of rare earth element resources.

## 2. Material and Methods

The composition values of the sintered Nd–Fe–B magnets were Nd_20.76_Pr_5.90_Ho_0.24_Gd_1.06_Fe_bal_M_1.4_ B_1.0_(M = Al/Co/Ga/Zr/Nb/Ni/Mn/, wt.%) (named N35). The main alloy Nd-Fe-B powder was prepared via strip-casting, hydrogen decrepitating, and jet milling. The auxiliary alloy Er_69_Fe_31_ was prepared through vacuum arc melting, followed by homogenization annealing at 800 °C for 14 days. The annealed alloy was then coarsely crushed and subsequently ball-milled using a planetary ball mill. The N35 powder and the auxiliary alloy powder were mixed under a nitrogen atmosphere. The uniformly mixed powder was then placed in a pulsed magnetic field moulding machine for directional moulding and isostatic pressing under a 2 T magnetic field and then sintered in a vacuum sintering furnace. The sintering temperature was 1050 °C for 5 h, which is lower than the melting point of the main phase (about 1185 °C) but much higher than the melting point of the Nd-rich phase (665 °C). During the sintering process, the Nd-rich phase and the auxiliary alloy Er_69_Fe_31_ melted into a liquid phase, which promoted the densification of the magnet. Then, the microstructure was further optimised using heat treatment to improve the magnetic properties. The heat treatment adopted a two-stage tempering process. The tempering process comprised two stages: first, high-temperature tempering at 880–960 °C for 2.5 h, followed by low-temperature tempering at 440–520 °C for 5 h.

NIM-10000H bulk rare earth permanent magnet non-destructive testing device (NIM-10000H, Beijing, China) was used to test the magnetic properties of the samples. The FA1104J electronic balance (Shanghai, China) was used to test the density of the samples. The melting point of Er_69_Fe_31_ was determined using differential scanning calorimetry (DSC, NETZSCH STA449F3, Bavaria, Germany) at 10 K/min. The grain orientation of the magnet powder samples was determined using X-ray diffraction (XRD, Empyrean PIXcel3D, Cu Kα, Almelo, The Netherlands). The composition phases were matched by the High Score Plus software (High Score Plus 3.0.5). Microstructure and the elemental distribution were observed using field-emission scanning electron microscopy (FESEM, Quanta 450 FEG, Hillsboro, OR, USA) with an energy-dispersive spectroscopy (EDS) attachment. The composition content of different phases can be obtained through point scanning, and the content variation and distribution of phases can be obtained by using line scanning and area scanning. In order to analyse the anti-corrosion characteristics of the target magnet, an electrochemical workstation (CHI660B, Chenhua, Shanghai, China) was used to measure the polarization curve to calculate the corrosion potential (*E_corr_*) and corrosion current density (*I_corr_*) of the magnets.

## 3. Results and Discussion

**Figure 1:**
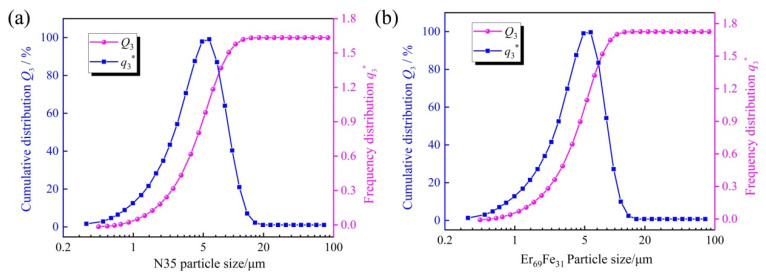
The cumulative distribution and frequency distribution of powder particles: (**a**) the sintered Nd-Fe-B magnet (N35), (**b**) Er_69_Fe_31_ alloy. q3* (q3lg)—Log-Normal Distribution.

The outcomes of the particle size assessment for Er_69_Fe_31_ alloy powders and Nd–Fe–B powders (N35) are shown in Figure 1a,b, respectively. A comparison of the particle size data of the two powders reveals the following results: the N35 magnet powder exhibits a surface mean diameter of 3.23 μm and a volume mean diameter of 4.90 μm, which are both bigger than the Er_69_Fe_31_ alloy powder with a surface mean diameter of 3.04 μm and a volume mean diameter of 4.59 μm. The findings are consistent with the experimental design, as smaller Er_69_Fe_31_ alloy powders have a higher propensity to access the grain boundary region. This enhanced accessibility facilitates a more effective function as a co-alloy through grain boundary reconstruction [19].

**Figure 2:**
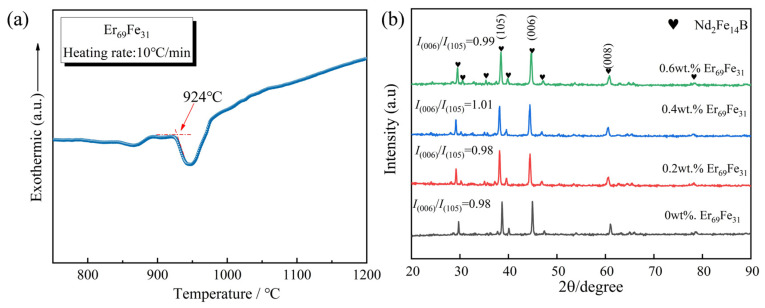
(**a**) Melting point of Er_69_Fe_31_ alloy; (**b**) XRD patterns of the magnets evaluated along the alignment plane with different Er_69_Fe_31_ additions.

Figure 2a shows that the melting point of the Er_69_Fe_31_ alloy is about 924 °C, exceeding the theoretical value of 915 °C [20]. During the process of sintering, a rise in the magnet’s temperature results in the preferential melting of the Nd-rich phase into a liquid state. Subsequently, the Er_69_Fe_31_ phase melts at the grain boundaries and fuses with the liquid grain boundaries. The grains of the main phase with the higher melting point remain as solid particles, thereby contributing to the optimisation of the grain boundaries [21]. Therefore, the Er_69_Fe_31_ can be used to assist the liquid phase sintering with the low melting point. The liquid crystalline boundary phase also infills internal magnet defects (holes, pores, and other imperfections), resulting in a smoother boundary layer morphology. Figure 2b shows the XRD diffraction patterns measured along the *C*-axis of the bulk magnets with different Er_69_Fe_31_ additions. The strong-intensity (00*l*) characteristic peaks indicated that an obvious *C*-axis crystallographic texture formed in all samples. Increases in the addition of Er_69_Fe_31_ (up to 0.4 wt.%) have been shown to result in a slight upward trend in the ratio of *I*_(006)_/*I*_(105)_, thereby validating the proposed interpretation [22,23]. The presence of Er_69_Fe_31_ alloys has been demonstrated to enhance grain boundary flow during the sintering process, thereby filling the internal defects of the magnet and aligning the main phase grains more closely along the *C*-axis. It has been demonstrated that this phenomenon consequently gives rise to a propensity to effect a change in the orientation of the magnet.

However, the (105) diffraction peak shows that there are a lot of stray peaks in the magnets that are not aligned with the main axis [24]. This phenomenon hinders the full orientation of the main phase grains. At present, it is widely accepted that the degree of magnet orientation is primarily influenced by two principal aspects. Firstly, the orientation pressing process involves the lateral forced flow of powder from both sides to the centre, deviating from the pressing direction. This results in the destruction of the orientation effect of the magnet particles, leading to the magnets being incompletely oriented [25]. Secondly, the grain growth and the capillary effect during liquid phase sintering affect the particle orientation inside the magnets [26].

**Figure 3:**
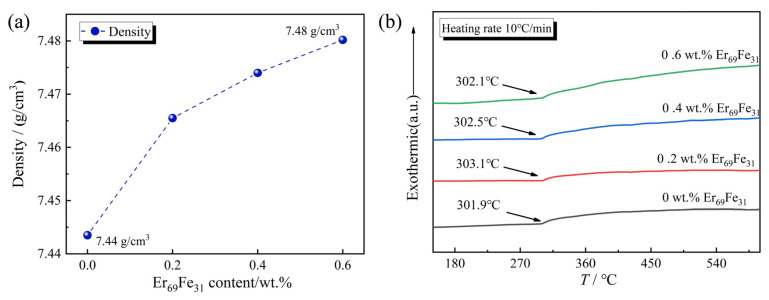
(**a**) Density of target magnets with different Er_69_Fe_31_ additions. (**b**) The Curie temperature of the target magnets with different Er_69_Fe_31_ additions.

Figure 3a shows that the density increases and achieves 7.48 g/cm^3^ at 0.6 wt.% Er_69_Fe_31_ addition compared with the initial magnet (0 wt.% Er_69_Fe_31_). Because the melting point of Er_69_Fe_31_ is much lower than the sintering temperature (1050 °C) [18], it will promote the distribution state of the grain boundary phases during the sintering process. Combined with the analyses in Figure 2a, it can be understood that the Er_69_Fe_31_ alloy melts into a liquid state at the grain boundary, which enhances the flow advantage of the liquid grain boundary phase during the sintering process. Figure 3b shows that the Curie temperature *T*_C_ increased to 303.1 °C when the Er_69_Fe_31_ addition reached 0.2 wt.%, which is 1.2 °C higher than the initial magnet. This proves that the introduction of the Er element does not deteriorate the Curie temperature *T*_C_ but maintains it compared with the initial magnet.

**Figure 4:**
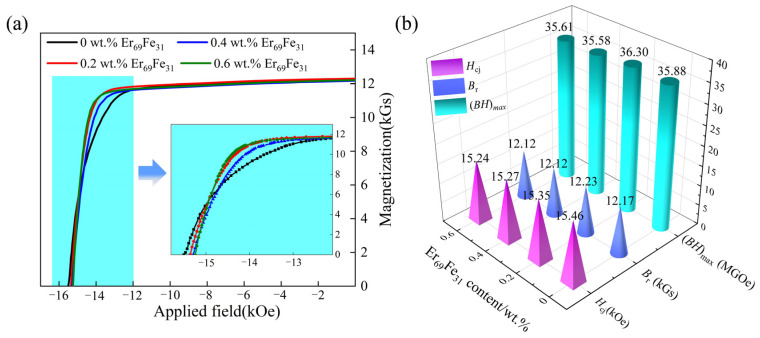
(**a**) Demagnetization curves of magnets containing different amounts of Er_69_Fe_31_, (**b**) variation in magnetic properties as a function of Er_69_Fe_31_ content.

As demonstrated in Figure 4, the magnetic properties of magnets vary with the addition of 0–0.6 wt.% Er_69_Fe_31_ alloy. By comparing the initial magnets (0 wt.% Er_69_Fe_31_), it was found that Er_69_Fe_31_ alloy addition resulted in a better optimisation of the demagnetisation curve in Figure 4a. However, it is evident that the coercivity *H*_cj_ exhibits a tendency to decrease marginally. This phenomenon can be attributed to several factors, including the uneven phase distribution at grain boundaries due to the presence of the Er_69_Fe_31_ alloy. This analysis also accounts for the interaction and restriction mechanisms during reverse magnetization, as well as the agglomeration behaviour of main phase grains. Compared to the initial magnet with *H*_cj_ of 15.46 kOe, *B*_r_ of 12.17 kGs, and (*BH*)_max_ of 35.88 MGOe, *H*_cj_ shows a gradually decreasing trend with Er_69_Fe_31_ addition increases from 0.2 wt.% to 0.6 wt.%, accompanied by slight changes of 0.11 kOe, 0.19 kOe, and 0.22 kOe, respectively. Although the *H*_cj_ is not significantly improved, the *B*_r_ is well maintained and increases from 12.17 to 12.23 kGs at 0.2 wt.% Er_69_Fe_31_ addition, resulting in a simultaneously increased (*BH*)_max_ from 35.88 to 36.30 MGOe.

**Figure 5:**
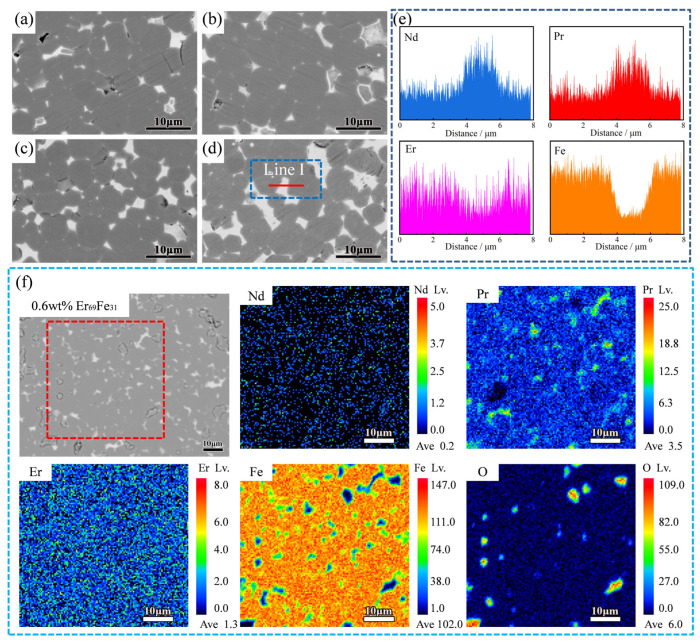
(**a**–**d**) Back-scattered SEM images of magnet treated using different Er_69_Fe_31_ additions ((**a**): 0 wt.%, (**b**): 0.2 wt.%, (**c**): 0.4 wt.%, (**d**): 0.6 wt.% Er_69_Fe_31_ addition); (**e**) corresponding line profile along the red line in (**d**) 0.6 wt.% Er_69_Fe_31_ addition; (**f**) EPMA images of the magnet treated by 0.6 wt.% Er_69_Fe_31_ (the corresponding Nd/Pr/Er/Fe/O mappings of red dotted line area).

From Figure 5a–d, the microstructure shows a substantial deterioration of the Nd-rich phase at the grain boundaries (white area) from 0.4 wt.% to 0.6 wt.% Er_69_Fe_31_ addition. The primary reason for the decrease in magnetic properties is the presence of a bulk Nd-rich phase in contact with the main phase. It can be mostly attributed to the non-magnetic phase amount of the introduced Er_69_Fe_31_ in grain boundaries, which causes a decrease in the relative volume fraction of the main phase [27]. It is also clear to see that Er elements tend to adhere to the edges of the main phase grains, and a small fraction of them enter the main phase, as shown in Figure 5e,f. This means that Er elements will replace some Nd elements like other rare earth elements such as Dy and Tb [28], forming (Er, Nd)_2_Fe_14_B with low coercivity, which leads to the deterioration of *H*_cj_ and the microstructure. However, the target magnet with 0.2 wt.% Er_69_Fe_31_ has fewer internal defects in Figure 3b than the initial magnet, which is accompanied by a slight increase in overall magnetic properties. Therefore, it is evident from the experimental results that the incorporation of Er_69_Fe_31_ alloys exerts a favourable influence on the magnetic properties of the target magnet, provided that the addition of these alloys is adequately regulated.

**Figure 6:**
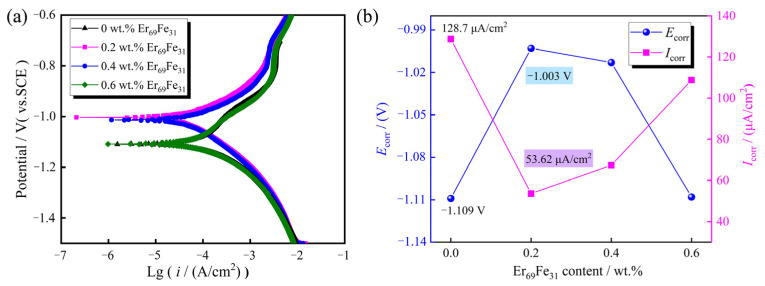
(**a**) Polarization curves of different target magnets in 3.5 wt.% NaCl aqueous solution; (**b**) corrosion potential (*E*_corr_) and corrosion current density (*I*_corr_) of different target magnets.

As illustrated in Figure 6, to explore whether the addition of Er_69_Fe_31_ alloy can improve the corrosion resistance of Nd–Fe–B magnets, the variation in anti-corrosion performance among different magnets in a 3.5 wt.% NaCl solution is demonstrated. Figure 6a presents polarization curves of the tested magnets in a 3.5 wt.% NaCl solution, while Figure 6b illustrates the trend of corrosion potential *E*_corr_ and corrosion current density *I*_corr_ of the target magnets that have been analysed. It can be found that the corrosion potential *E*_corr_ shifts from −1.109 V to −1.003 V at 0.2 wt.% Er_69_Fe_31_ addition compared to the initial magnet. The increase in the corrosion potential *E*_corr_ also indicates that the thermodynamic properties of the target magnet are improved after the addition of the Er_69_Fe_31_ alloys, resulting in a decrease in the corrosion of the target magnet [29]. Meanwhile, the corrosion current density *I*_corr_ is reduced from 128.7 μA/cm^2^ to 53.62 μA/cm^2^ at 0.2 wt.% Er_69_Fe_31_ addition, which clearly shows that the introduced Er_69_Fe_31_ alloy can effectively reduce the corrosion rate of the target magnet.

**Figure 7:**
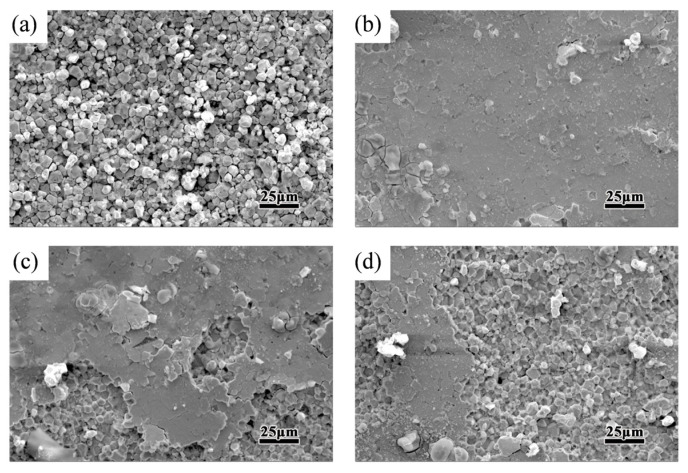
(**a**–**d**) Corrosion morphologies of target magnets with different amounts of Er_69_Fe_31_ after polarization curve test ((**a**): 0 wt.%, (**b**): 0.2 wt.%, (**c**): 0.4 wt.%, (**d**): 0.6 wt.% Er_69_Fe_31_ addition).

Figure 7 shows the magnets’ corrosion morphology after the polarization curve test. It can be found that the grain boundary phase of the initial magnet (Figure 7a) was corroded seriously, and the matrix phase became an independent grain, accompanied by some shedding phenomena. Combined with the analysis of the polarization curve test in Figure 6, the target magnet has the smallest corrosion area, and the best corrosion resistance is achieved at 0.2 wt.% Er_69_Fe_31_ addition. A small amount of Er elements dispersed in the grain boundary area that have a higher electrode potential than Nd elements, which can hinder the propagation of corrosion pathways in the grain boundary area and improve the anti-corrosive performance [18]. However, as the Er_69_Fe_31_ alloy addition continues to increase, the grain shedding phenomenon becomes more obvious, as shown in Figure 7c,d. This is due to the increased volume of Nd-rich regions, which elevates the probability of corrosion-prone sites in the magnet, consequently reducing its corrosion resistance. This indicates that moderate Er content can effectively slow the magnet’s corrosion rate.

**Figure 8:**
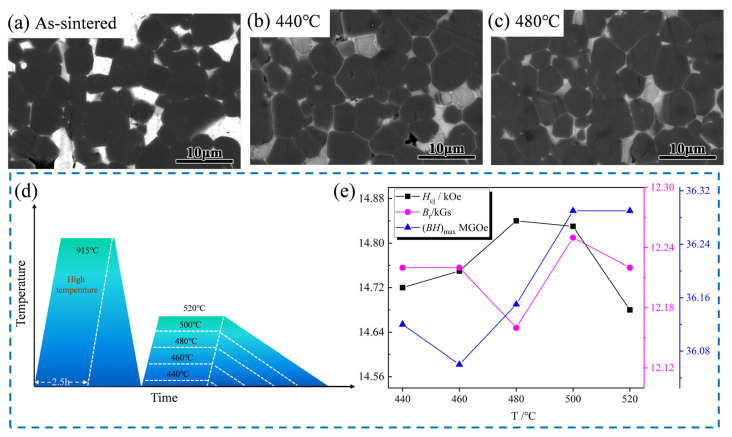
The change in microstructure and magnetic properties of the target magnet with low-temperature tempering under the first heat treatment with 0.4 wt.% Er_69_Fe_31_ addition (1st) ((**a**) sintered state, (**b**) 915 °C × 2.5 h + 440 °C × 5 h, (**c**) 915 °C × 2.5 h + 480 °C × 5 h; (**d**) schematic diagram of heat treatment at low temperatures; (**e**) room-temperature magnetic property change curve for low-temperature heat treatment).

Figure 8 displays the microstructure and magnetic properties of magnets treated by 0.4 wt.% Er_69_Fe_31_ at low-temperature tempering under the first heat treatment (the detailed heat treatment process at low-temperature tempering under the first heat treatment can be seen schematically in Figure 8d). From the sintered state in Figure 8a, it can be seen that the microstructure without heat treatment is relatively disordered, Nd-rich phases form blocky precipitates at grain boundaries, and numerous main phase grains are in direct contact. Therefore, there are no continuous and uniform thin Nd-Rich phases, which causes poor wettability between the main phases in this distribution state [30]. This reasonably explains why the sintered magnet has low magnetic properties. Compared to the sintered state magnets (Figure 8a), Figure 8b,c reveal that the number of massive grain boundary phases (white area) in the internal microstructure of the magnets is reduced, while the number of striped grain boundary phases is increased at 480 °C. Significant enhancements and optimisations were observed in both the magnetic property values and the microstructure. This phenomenon can be attributed to the dilution of the volume fraction in the primary phase (grey-black area) due to the excess of non-magnetic phase grain boundary phases. This finding is in accordance with the *H*_cj_ at both temperatures (440 °C: 14.72 kOe < 480 °C: 14.84 kOe), suggesting that low-temperature tempering exerts a minor effect on the magnetic properties and microstructure.

As demonstrated in Figure 8e, the magnetic properties of the target magnet vary with increasing low-temperature tempering temperatures. When considered in conjunction with the comprehensive magnetic property data presented in Table 1, the target magnet *B*_r_ experiences minimal fluctuations between 460 °C and 520 °C, remaining within the range of 12.16–12.25 kGs. The *H*_cj_ ranges from 14.68 kOe to 14.84 kOe, with a maximal value of 14.84 kOe attained at 480 °C. However, the (*BH*)_max_ obtained the maximum value of 36.29 MGOe at 500 °C and 520 °C. From the perspective of industrial production costs and comprehensive magnetic property evaluation, the target magnets with 0.4 wt.% Er_69_Fe_31_ additions in the 915 °C × 2.5 h + 480 °C × 5 h heat treatment process exhibited optimal magnetic properties. Therefore, the low-temperature tempering process has been shown to enhance the magnetic properties of the target magnets to a certain extent, both in comparison to the sintered magnets and to the same set of low-temperature tempered target magnets.

**Figure 9:**
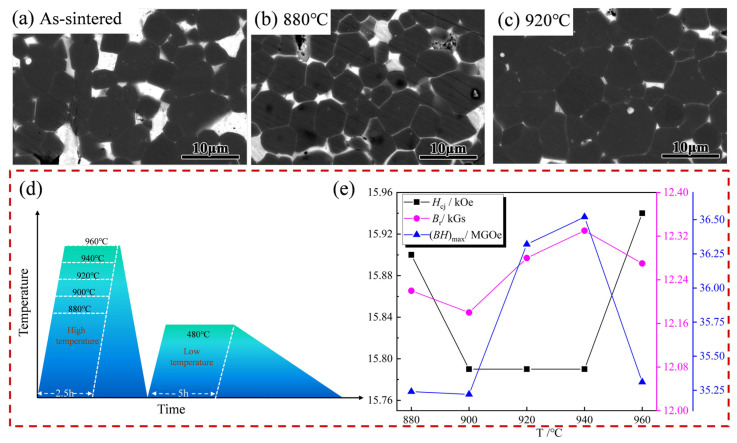
The change in microstructure and magnetic properties of the target magnet with high-temperature tempering under the second heat treatment cycle with 0.4 wt.% Er_69_Fe_31_ addition (2nd) ((**a**) sintered state, (**b**) 880 °C × 2.5 h + 480 °C × 5 h, (**c**) 940 °C × 2.5 h + 480 °C × 5 h, (**d**) schematic diagram of heat treatment at high temperatures, (**e**) room-temperature magnetic property change curve for high-temperature heat treatment).

Figure 9 shows the microstructure and magnetic property change curves of high-temperature tempering with 0.4 wt.% Er_69_Fe_31_ addition under the second-cycle heat treatment (the detailed heat treatment process at high temperature under the second-cycle heat treatment can be seen schematically in Figure 9d). Figure 9b,c reveal significant performance improvements in heat-treated magnets due to microstructural optimisation achieved through high-temperature tempering. This microstructural behaviour contrasts sharply with the as-sintered state shown in Figure 9a. In Figure 9c (940 °C) and Figure 9b (880 °C), the presence of a bulk grain boundary phase (white area) gradually diminishes, while the grain boundary phase of the reticular structure concomitantly increases, enveloping the main phase (grey-black area) grains. This idealised microstructure has the capacity to enhance the wettability between the primary phases, while concurrently functioning as an isolating agent for the primary phases, thereby leading to a substantial enhancement in the coercivity [31].

From Figure 9e and Table 2, the magnetic properties of the target magnets were significantly enhanced in the second cycle compared to the first cycle. The *B*_r_ of the target magnet fluctuates within a small range of 12.18 to 12.33 kGs, while the *H*_cj_ fluctuates within a range of 15.79 to 15.94 kOe, reaching a maximum value of 15.94 kOe at 960 °C. It is evident that the *H*_cj_ increases by 16.7%relative to the as-sintered magnet. The (*BH*)_max_ of 36.38 MGOe was obtained at 940 °C. This result demonstrates the efficacy of the meticulously designed secondary-cycle heat treatment in enhancing the magnetic properties of magnets with 0.4 wt.% Er69Fe31 addition. The combined magnetic properties (*H*_cj_ + (*BH*)_max_) of the target magnet are 51.14, 51.01, 52.11, 52.31, 51.25 from 880 °C to 960 °C, respectively. With regard to the overall magnetic properties, the target magnet with 0.4 wt.% Er_69_Fe_31_ additions demonstrates optimal magnetic properties at 940 °C. When taking into consideration the financial implications of production, the most cost-effective heat treatment for the target magnet is determined to be 940 °C × 2.5 h + 480 °C × 5 h. It was also determined that this optimum high-temperature tempering temperature is in close proximity to the melting point of the Er_69_Fe_31_ alloy (924 °C). It can be hypothesised that this is attributable to the fact that the Er_69_Fe_31_ alloy has not yet undergone full reaction with the grain boundary phases during the initial heat treatment. Therefore, the elevated temperature stage of the second cycle of the heat treatment will facilitate further reaction, thereby optimising the microstructure of the magnets through liquid phase sintering.

## 4. Conclusions

In this study, the effects of the Er_69_Fe_31_ on the magnetic properties, microstructure distribution and corrosion resistance of the target magnets were mainly investigated using the grain boundary addition technology. (1) The coercivity *H*_cj_ decreases by 0.11 kOe compared with the initial magnet, a preferable magnetic performance obtained for *B*_r_ of 12.23 kGs and (*BH*)_max_ of 36.30 MGOe upon 0.2 wt.% Er_69_Fe_31_ addition. (2) As analysed using the EDS technique, Er elements rarely enter the main phase grains, a small part of which is dispersed in the grain boundary region and most of which accumulates in the main phase grains to form a low *H*_A_ (Er, Nd)_2_Fe_14_B shell structure, resulting in a decrease in coercivity. (3) The corrosion resistance of target magnets after the grain boundary reconstruction of the Er_69_Fe_31_ alloy is improved at 0.2 wt.%. The corrosion potential (*E*_corr_) is increased from −1.109 V to −1.003 V, and the corrosion current density (*I*_corr_) is reduced from 128.7 μA/cm^2^ to 53.62 μA/cm^2^. (4) Selecting 0.4 wt.% Er_69_Fe_31_ added sintered magnets, we studied the effects of low-temperature tempering in the first heat treatment and high-temperature tempering in the secondary cyclic heat treatment. The results show that secondary cyclic heat treatment greatly improves the magnet performance and microstructure compared to the first treatment. The optimal process is 940 °C × 2.5 h + 480 °C × 5 h.

## Figures and Tables

**Table 1:** Magnetic properties of target magnets with 0.4 wt.% Er_69_Fe_31_ addition at different low-temperature tempering temperatures.

Sample	High Temperature/°C	Low Temperature/°C	*H*_cj_ (kOe)	*B*_r_ (kGs)	(*BH*)_max_ (MGOe)
Sintered state	—	—	13.65	12.10	31.58
1	915 °C × 2.5 h	440 °C × 5 h	14.72	12.22	36.12
2	915 °C × 2.5 h	460 °C × 5 h	14.75	12.22	36.06
3	915 °C × 2.5 h	480 °C × 5 h	14.84	12.16	36.15
4	915 °C × 2.5 h	500 °C × 5 h	14.83	12.25	36.29
5	915 °C × 2.5 h	520 °C × 5 h	14.68	12.22	36.29

**Table 2:** Magnetic properties of 0.4 wt.% Er_69_Fe_31_ target magnets at different high-temperature tempering temperatures in secondary-cycle heat treatment.

Sample	High Temperature/°C	Low Temperature/°C	*H*_cj_ (kOe)	*B*_r_ (kGs)	(*BH*)_max_ (MGOe)
Sintered state	—	—	13.65	12.10	31.58
1	880 °C × 2.5 h	480 °C × 5 h	15.90	12.22	35.24
2	900 °C × 2.5 h	480 °C × 5 h	15.79	12.18	35.22
3	920 °C × 2.5 h	480 °C × 5 h	15.79	12.28	36.32
4	940 °C × 2.5 h	480 °C × 5 h	15.79	12.33	36.52
5	960 °C × 2.5 h	480 °C × 5 h	15.94	12.27	35.31

## Data Availability

The original contributions presented in this study are included in the article. Further inquiries can be directed to the corresponding authors.
